# Persons ‘never treated’ in mass drug administration for lymphatic filariasis: identifying programmatic and research needs from a series of research review meetings 2020–2021

**DOI:** 10.1093/inthealth/ihad091

**Published:** 2024-09-05

**Authors:** Molly A. Brady, Emily Toubali, Margaret Baker, Elizabeth Long, Caitlin Worrell, Kapa Ramaiah, Patricia Graves, T. Deirdre Hollingsworth, Louise Kelly-Hope, Diana Stukel, Bhupendra Tripathi, Arianna Rubin Means, Sultani Hadley Matendechero, Alison Krentel

**Affiliations:** a Department of Global Health, RTI International, Washington, DC 20008, USA; b Neglected Tropical Diseases Division, Office of Infectious Disease, Bureau for Global Health, United States Agency for International Development, Washington, DC 20547, USA; c Georgetown University, Washington, DC 20057, USA; d Neglected Tropical Diseases Support Center, Task Force for Global Health, Decatur, GA 30030, USA; e Division of Parasitic Diseases and Malaria, U.S. Centers for Disease Control and Prevention, Atlanta, GA 30329, USA; f Department of Epidemiology, Swiss Tropical and Public Health Institute, Basel 4051, Switzerland; g Faculty of Science, University of Basel, Basel 4001, Switzerland; h Consultant, Lymphatic Filariasis Epidemiologist, Pondicherry, India; i College of Public Health, Medical and Veterinary Sciences and WHO Collaborating Centre for Vector-Borne and Neglected Tropical Diseases, James Cook University, Nguma-bada Campus, Cairns, QLD 4870, Australia; j Big Data Institute, Li Ka Shing Centre for Health Information and Discovery, University of Oxford, Oxford, OX3 7LF, UK; k Liverpool School of Tropical Medicine, Pembroke Place, Liverpool, L3 5QA, UK; l University of Liverpool, Institute of Infection, Veterinary and Ecological Sciences, Brownlow Hill, Liverpool, L2 5RF, UK; m Act to End Neglected Tropical Diseases West, Department of Global Health and Population, FHI 360, Washington, DC 20009, USA; n Bill and Melinda Gates Foundation, India Country Office, New Delhi 110067, India; o Department of Global Health, University of Washington, Seattle, WA, USA; p Kenya National Public Health Institute, PO Box 30016-00100, GPO Nairobi, Kenya; q School of Epidemiology and Public Health, University of Ottawa, 600 Peter Morand Drive, Ottawa, ON K1G 5Z3, Canada; r Bruyère Research Institute, Ottawa, ON K1N 5C8, Canada

**Keywords:** adherence, compliance, elimination, equity, filariasis, mass drug administration

## Abstract

As neglected tropical disease programs rely on participation in rounds of mass drug administration (MDA), there is concern that individuals who have never been treated could contribute to ongoing transmission, posing a barrier to elimination. Previous research has suggested that the size and characteristics of the never-treated population may be important but have not been sufficiently explored. To address this critical knowledge gap, four meetings were held from December 2020 to May 2021 to compile expert knowledge on never treatment in lymphatic filariasis (LF) MDA programs. The meetings explored four questions: the number and proportion of people never treated, their sociodemographic characteristics, their infection status and the reasons why they were not treated. Meeting discussions noted key issues requiring further exploration, including how to standardize measurement of the never treated, adapt and use existing tools to capture never-treated data and ensure representation of never-treated people in data collection. Recognizing that patterns of never treatment are situation specific, participants noted measurement should be quick, inexpensive and focused on local solutions. Furthermore, programs should use existing data to generate mathematical models to understand what levels of never treatment may compromise LF elimination goals or trigger programmatic action.

## Introduction

In 2020, the World Health Organization (WHO) introduced the 2021–2030 Neglected Tropical Disease (NTD) Roadmap, which outlines disease-specific goals for 20 NTDs.^1^ Preventive chemotherapy for NTDs, often delivered through mass drug administration (MDA), treats people in endemic areas regardless of their infection status and is a core intervention used by several NTD programs to achieve disease control or elimination.^1,2^ Published NTD models demonstrate that the likelihood of reaching elimination through MDA is associated with higher coverage,^3^ less clustering of persons missing treatment^4^ and fewer persons never treated.^5^

NTDs are defined by their burden on the poorest and most marginalized populations. The plan to end poverty, laid out in the 2030 Agenda for Sustainable Development, includes as a measurable target, ‘ending neglected tropical disease epidemics by 2030’ (target 3.3), alongside ‘achieving universal health coverage (UHC) with access to quality services and medicines for all’ (target 3.8).^6^ The first WHO/World Bank report on tracking UHC ties these two targets together when it states that measuring preventive chemotherapy treatment coverage for NTDs is ‘key to ensuring that the diseases of the least well-off are being prioritized from the very beginning of the path towards UHC’.^7^

The WHO has issued guidelines for measuring treatment coverage through routine administrative reporting, post-MDA coverage evaluation surveys and rapid estimates done by supervisors during MDA.^8–10^ To date, these have focused on measuring the percentage of the target and at-risk populations treated in the last MDA. Programs targeting lymphatic filariasis (LF)—a mosquito-borne parasitic disease—use reported annual MDA coverage to determine readiness to conduct impact surveys, which in turn determine whether MDA can be stopped. However, there is little focus on identifying persons never treated, their potential role in driving continued transmission and understanding their sociodemographics, geographic distribution, infection status and reason(s) for not being treated, among other factors.

As elimination programs increasingly reach the endgame and prepare for the post-elimination phase, there is concern that people who have never been treated could contribute to continued transmission, posing a barrier to elimination. Previous research has suggested that the size and distribution of the population never treated with MDA may be an important issue—as ongoing transmission in a large population could threaten achievement of elimination targets—and one that has not been sufficiently addressed within the research community.^11–15^

To address this critical knowledge gap, a series of expert meetings was held and included participation from a variety of organizations represented by the authors of this article, among others. This article summarizes the information shared and proposes next steps, including defining metrics, expanding routine data collection and an operational research agenda. While we focus on LF, lessons from this NTD are applicable to other diseases and public health areas.

### Research links meeting series

The Coalition for Operational Research on Neglected Tropical Diseases and the Improving Community Health Outcomes through Research, Dialogue and Systems Strengthening (iCHORDS) community of practice hosted a series of four virtual meetings from December 2020 to May 2021 designed to explore and compile expert knowledge on persons never treated in LF MDA programs. Invited participants had previous research experience in this area and/or were exploring this through routine programmatic monitoring and evaluation. The meetings explored four questions: the number and proportion of people never treated, their sociodemographic characteristics, their infection status and the reasons why they were not treated.

The first three meetings were held with 30 experts from LF national programs, the WHO, research institutions, implementing partners and donors. A smaller group met to discuss and propose more appropriate and inclusive terminology. All meeting information was then packaged, shared and discussed in a public webinar with 174 participants from 47 countries. This article presents the consolidated proceedings of these meetings and proposed next steps.

## Terminology

In the literature, the phenomena of frequently not swallowing or never swallowing MDA medicines across any round has multiple terms, including systematic non-compliance, persistent non-compliance, systematic non-adherence and semi-systematic non-compliance (Table 1). These terms have generally referred to those intentionally refusing (e.g. due to fear or lack of perceived need) and those not given the opportunity to take the treatment (e.g. due to ineligibility, lack of knowledge of the MDA or never having been offered MDA). Conversely, some literature captures the inverse of never taken by noting which people have ever swallowed MDA tablets.^16^

In the broader health literature, compliance is usually defined as the act of an individual conforming to professional recommendations with regards to prescribed dosage, timing and frequency of an intervention^24^ or the extent to which a patient acts in accordance with the prescribed interval and dose of a regimen.^25^ Many in the NTD community have indicated that the term ‘compliance’ is neither adequate nor appropriate to illustrate swallowing LF tablets during MDA because it assumes the individual has no agency in the decision and risks oversimplifying the complex programmatic and individual reasons a person may not take medicines. The medical literature has also used the terms ‘medicine persistence’ and ‘concordance’ to describe compliance. Medicine persistence illustrates the duration of time from initiation to discontinuation of therapy,^25^ while concordance infers that the prescriber and patient must come to an agreement about the regimen the patient will take.^26^ An alternative term, ‘adherence’, describes the extent to which a person’s behaviour corresponds with the agreed recommendations from a healthcare provider.^27^ None of these terms adequately describe whether an individual receives treatment and do not specify either intentional refusals and/or unintentional reasons for missing treatment.

After soliciting additional input from stakeholders, the term ‘never treated’ was suggested to capture those individuals who self-report that they have never ingested tablets during any round of LF MDA. ‘Never treated’ does not put the onus for taking LF tablets solely on either the program or the recipient. In addition, it has the benefit of being easily understood and translated into various languages. The use of ‘treatment’ is consistent with nomenclature used in the WHO NTD 2030 Roadmap, which lists a target of 90% reduction in the number of people requiring treatment for NTDs.^1^ The WHO’s Joint Application Package (JAP) uses similar terminology in its register to capture ‘reasons for non-treatment’ at the peripheral level.^28^ In this article we will use the terminology ‘never treated’ to represent those individuals who either self-report never treatment or who have been identified through registers to have not taken any LF tablets during any MDA rounds.

## What we already know

### Proportion of persons never treated

Experts shared experiences that showed wide variation of results (within and between countries) in the proportion of people who reported having never been treated in LF MDA. This is illustrated in the summary of results from published studies that were presented in the meeting series (Table 2). These data were collected using a variety of methods, with different sample sizes and from different populations (different ages, and while some included only those eligible for treatment in the denominator, others included all respondents). These differences pose challenges for comparing results across settings and underpin the need for standardized terminology and metrics.

### Characteristics of persons never treated

Noting the small number of published studies presented that included characteristics of persons never treated,^12,15^ experts also shared information from ongoing work and unpublished research and from national NTD program routine data. Based on published studies and unpublished data, they hypothesized that there may be higher proportions of persons never treated among hard-to-reach populations (including those living in very remote areas, migrants and urban centres), those with limited awareness about LF and MDA and those who did not know if others in their household took the LF medicines. Trends such as systematic differences among world regions and by MDA drug regimen emerged. Meeting participants reported different associations of never treated by age group and sex. For example, in some settings, more men than women were never treated, perhaps due to not being home because of their occupation, while in other settings it was women who were more often never treated, possibly related to repeated pregnancies across MDA rounds and thus not being eligible to receive treatment.

### Reasons for never treatment

Experts had limited information to share on why people were never treated in MDA, as most of the data pertained to why people were not treated during the most recent round of MDA offered. Reported reasons for not being treated during the last MDA round included ineligibility, absence, fear of side effects and the perception of not being at risk; these have been documented in previous literature reviews for LF and other NTDs.^19,34,35^ While these reasons are commonly accepted, they require more investigation, e.g. absence could imply not being home at the time of day the drug distributor came, intentional avoidance or travel out of the district for the duration of the MDA campaign. Similarly, fear of side effects could imply the desire to take tablets after eating or at night, reluctance due to a lack of follow-up care for side effects or fear of death from the medicines due to rumours. These reasons will vary culturally across contexts and may also be directly linked to the effectiveness of the social mobilization campaign. Variations in how questions were asked and how data were analysed resulted in reasons being grouped differently, making comparing results across studies difficult.

Experts discussed the challenges in capturing explanations as to why people were never treated. These reasons would likely vary for the same person from year to year and open to recall bias and the relative importance of each reason would be difficult to determine.

### Association between never treated and infection status

Having groups of never-treated people, regardless of who they are and why they were not treated, is of concern if they are infected and therefore at personal risk of clinical disease and potentially contributing to ongoing transmission. In Egypt, after completing five rounds of MDA, with overall coverage >85%, it was found that 7.4% of the study population was never treated in any of the five rounds of MDA. Infection rates, as measured by microfilaraemia and antigenaemia tests, were statistically significantly higher in the groups who reported taking zero or one round of treatment compared with those who took two or more.^36^ Similar results were found in one American Samoa study where 6% of persons were never treated and those who had ever been treated in MDA had a lower odds of infection compared with those never treated in multiple regression analysis (odds ratio [OR] 0.39, p = 0.04).^29^ However, these results were not replicated in two other American Samoa studies with higher percentages (31% and 58%) of persons reporting to have been never treated.^30,31^ A study in Samoa found higher antigen prevalence (5.8%) among participants who reported never taking MDA compared with those who reported taking MDA at least once (4.9%), but the difference was not statistically significant.^32^ In Myanmar, significantly higher infection rates among the never treated in a univariate analysis did not hold when other factors were controlled for in a multiple regression analysis.^37^

## Suggestions to strengthen measurement of never treatment

### Create standard indicators on frequency of treatment

Contributing to this general lack of information on never-treated persons—number, characteristics, why they were never treated and their role in contributing to continued transmission—is the lack of a standardized indicator and measurement in routinely collected programmatic data or in research studies (Table 3). During analysis, some researchers grouped ‘treated zero or once’ versus ‘treated twice or more’, while others grouped ‘never treated’ versus ‘treated once or more’. Researchers have also used variations of survey questions to understand who has never been treated. One of the most common questions used was ‘including this year, how many times have you taken the medicines for LF?’ with possible responses of ‘never’, ‘one time’, ‘two or more times’. This indicator has been validated through use in population-based surveys, in acceptability studies as well as in some national programmatic data collection.^12,15,33^

Because studies have shown that two or more rounds of annual diethylcarbamazine plus albendazole or twice a year albendazole clears filarial infections significantly faster than zero or one round^11,38^ and that just one round of triple drug therapy almost totally clears microfilaraemia,^39^ experts recommended reporting results for both ‘never treated’ and ‘treated once’ in addition to treatment in two or more rounds. Further recommendations for measurement included disaggregating data by age and sex. This will not account for the potential to routinely miss certain people in surveys and so considerations for weighting or adjusting the timing of surveys also should be considered.

### Modify existing tools

There are different quantitative and qualitative tools that LF programs use routinely to assess disease prevalence, MDA coverage and implementation methods (Table 3). Meeting participants identified opportunities to modify the design, use and analysis of these existing tools to increase knowledge on who is not treated, including the never treated. Participants reported from experience that it is feasible to add questions on the frequency of past treatment to pre-transmission assessment surveys (TASs), coverage evaluation surveys and the Supervisor’s Coverage Tool (SCT). Pre-TASs have the advantage of linking infection status with never-treatment data; however, it collects data from two or three high-risk sites within a district so is not representative of the entire district. Participants also agreed that a standardized list of options to record reasons for non-treatment is needed to allow for comparing and synthesizing data collected across studies and countries. For example, currently the term ‘ineligible’ does not differentiate between those contraindicated for treatment and those who are misidentified as ineligible, but that distinction is important in planning programmatic responses. Similarly, there would also be value in providing options that provide more nuance to answers like ‘absent’ or ‘side effects’. Clear categorization of these responses is needed to better link reasons for never being treated with potential solutions. At a minimum, results should be disaggregated by people who were never treated due to issues with program reach (unintentional) or due to individual refusal (intentional). Qualitative research may also need to be used and current operational research is under way to explore potential approaches and methods.

Other adjustments currently being piloted by programs and research studies include oversampling specific populations of interest (e.g. migrants, youth, males) and collecting information on other variables that would elicit programmatically actionable information, such as levels of trust in drug distributors, health behaviour influencers in the community and migration patterns. Suggested modifications also include adding diagnostic tests to enable linking treatment history to infection status and collecting georeferenced data to conduct geospatial analysis that visually represents associations between multiple variables.

### Ensuring the never treated are not missing from other data collection

One concern expressed by several participants is that there may be selection bias inherent in the design of existing tools that needs to be explored and, if necessary, addressed. The same people missed in surveys designed to estimate coverage and infection prevalence may also be missed by MDA, potentially impacting the coverage and infection prevalence estimates produced. For example, in a study in American Samoa, 97.5% respondents were of the majority Samoan ethnic group, although census data showed that 15% of the population in the area were from non-Samoan ethnic groups.^30^ The use of proxy responders, permitting a household member to respond on behalf of someone who was not home at the time of the survey or could not answer for themselves, was also questioned. A recent analysis of NTD coverage surveys in three countries hypothesized that proxy responses may lead to an inflation of surveyed drug coverage.^40^ Thus it was recommended that analyses of never treatment exclude proxy responses. Overall, ensuring never treated are not missing from data collection requires further exploration—both programmatically by adjusting the time of day or week or year that both MDA and the surveys are conducted and with operational research.

## Adapting to reach those who have never been treated

Ultimately data collected on persons never treated needs to be used to adapt strategies for distributing the medicines. These strategies will need to be specific to the local context and will likely vary according to geography, such as urban versus rural setting, and population groups involved, such as undocumented migrants, transient labour forces or other marginalized groups. In previous research, data collected on people who were never treated was useful for tailoring and refining MDA strategies to improve reach and strengthen equity in groups where coverage had been previously low.^12,33^ This may include social mobilization approaches that are adapted using gender-specific messages and young people as behaviour change agents (personal communication arose from some of the experts). Motivating drug distributors to identify individuals who have never been treated during the MDA was proposed as a response to never treatment in Indonesia.^12^ In Myanmar, recommendations were made to change the timing of treatment to align with when people are at home and ensuring resources were available for mop-up.^37^ Finally, other public health programs, such as immunization campaigns, may be helpful to LF programs in sharing techniques they have used to reach those never treated, such as when to use fixed locations versus house-to-house distribution, extending personal invitations to participate from health workers and strategic use of media.

## Conclusions and next steps

The expert review presented here has confirmed there is a lack of evidence and understanding about the never treated and their impact on LF elimination programs. Little is known about the proportion of persons never treated, their infection status, their demographic profiles, reasons for not being treated and effective programmatic responses. There is mounting evidence that people never taking drugs (or taking them only once) are slower at clearing infection, but little is published on the impact of groups who have never taken LF treatment on ongoing transmission. If persons never treated are found to be high infection reservoirs, tackling this challenge will be critical to reaching NTD elimination targets.

We recognize that patterns of never treatment are context specific—often down to the village level. Measuring the issue of never treated therefore needs to be quick and inexpensive, with an aim to adapt solutions to local contexts. Given the 2030 elimination goals for LF, the need is urgent. Meeting participants noted that this can be done most feasibly and efficiently by modifying existing programmatic tools that are already in wide-scale use. Synthesis of data and learning across settings is also important to identify common challenges and solutions that can be tried in different settings. Standardization of questions and indicators will be crucial for cross-site analysis. Furthermore, as data are collected on those who are never treated, these data need to be utilized to improve mathematical models that help determine what levels of never-treatment risk elimination goals should trigger programmatic action. Specific opportunities for further analysis and operational research proposed have been captured in Boxes 1 and 2, some of which are related to more general coverage issues and some of which are specific to never treatment. To move this agenda forward, a core group of meeting participants volunteered to develop data collection and analysis standard operating procedures to be shared widely on the iCHORDS website (www.ichords.org) for use and real-time feedback by national programs, implementing partners and researchers.

Evidence regarding populations who are never treated, their infection status and why they have not taken treatment will strengthen programmatic responses to the broader issue of low MDA treatment coverage. New knowledge generated from these next steps will improve access to MDA and ultimately increase coverage towards more universal healthcare.

## Attendees of the research review meetings

Presenters: Molly Brady (RTI International), Patricia Graves (James Cook University), Deirdre Hollingsworth (University of Oxford), Louise Kelly-Hope (Liverpool School of Tropical Medicine), Alison Krentel (University of Ottawa and the Bruyere Research Institute), Bhupendra Tripathi (Bill & Melinda Gates Foundation India Country Office), Diana Stukel (FHI 360) and Gary Weil (DOLF Project, Washington University at St Louis).

Attendees: Bill & Melinda Gates Foundation: Molly Mort, Bhupendra Tripathi and Rosalyn Yeary; FCDO: Dirk Mueller; FHI360: Diana Stukel; HELLP at Emory University: Lee Wilkers; James Cook University: Patricia Graves; Liverpool School of Tropical Medicine: Louise Kelly-Hope; University of Oxford: Deirdre Hollingsworth; NTD Support Center at Task Force for Global Health: Katie Gass, Pat Lammie and Elizabeth Long; Noguchi Memorial Institute-Ghana: Dziedzom de Souza; RTI International: Margaret Baker and Molly Brady; Sightsavers: Rogers Nditanchou; US Agency for International Development: Emily Toubali; US Centers for Disease Control and Prevention: Tara Brant and Caitlin Worrell; University of Ottawa and Bruyère Research Institute: Leshawn Benedict and Alison Krentel; University of Washington: Arianna Rubin Means; Washington University at St. Louis: Peter Fischer and Gary Weil; World Health Organization: Jonathan King, Denise Mupfasoni, Kapa Ramaiah, Ronaldo Carvalho Scholte, Anthony Solomon and Aya Yajima.

## Figures and Tables

**Table 1.** Previous definitions of systematic non-compliance

Definitions used	Country (reference)
Individuals who systematically do not adhere to treatment, over a number of treatment rounds	Non-specific^17^
Individual who never attended any rounds of MDA/people who are never treated in any rounds of MDA	Non-specific/modelling paper^3^
People who persistently refuse or do not ingest the antifilarial medications over the course of an MDA program	Indonesia^18,19^
People who have never participated in an MDA	Haiti^20^
People who miss all rounds of MDA	Egypt^11^
The proportion of people who never took the medicines during the three distributions of MDA	Haiti^21^
Individuals receive treatment in every round but never swallow the tablets (persistent non-compliers)	India^22^
Proportion of the population who repeatedly miss or refuse MDA	WHO-WER^23^

**Table 2.** Proportion of people never treated in LF MDA, from published literature

Country	Study year	Location	Tool	Age group (years)	Sample size	Previous MDA rounds, n	Never treated, %
American Samoa^29^	2007	Urban and rural	Randomly selected household survey	≥2	1881	7	6
American Samoa^30^	2014	Urban and rural	Worksite study	≥15	496	7	31
American Samoa^31^	2016	Urban and rural	30 randomly selected villages	≥8	2507	7	58
Samoa^32^	2018	Urban and rural	Population-based survey with 35 clusters	≥2	4420	8–10, 9–11	14 (pre-2018); 3 (including 2018)
Fiji^33^	2019	Rural	Community-based study	>14	300	>10	15
Myanmar^16^	2015	Peri-urban and rural	Population-based household survey	>1	1014	6	19
India^33^	2019	Peri-urban	Community-based study	>14	397	12	85
India^22^	1994–2000	Rural	Prospective study in 10 villages	Individuals >15 kg	18 415	6	3.5–4 of those eligible in all rounds
Indonesia^12^	2013–2014	Urban and rural	Community-based study	>15	806	5	19 (rural) and 24 (urban)
Egypt^11^	2000–2006	Rural	Coverage evaluation survey	>5	1064	5	7
Haiti^33^	2019	Peri-urban	Community-based study	>14	407	8	25
Haiti^20^	2008	Peri-urban	Population-based household survey	>5	455	7	24
Guyana^15^	2021	Peri-urban	Community-based pre-MDA study	>18	451	3	21

**Table 3.** Summary of methodological issues and suggested approaches

Issue	Description	Suggestions
Measuring ‘never treated’ (NT)	There is no standardized indicator used within programmatic data and research for measuring NT	Use NT indicator: How many times have you taken the medicines for LF? with responses of never, one time or two or more times. Use age and sex standardization of the NT indicator. Further validate the proposed indicator against treatment registers, where available.
Adapting design and use of existing tools	Tools that are widely used rarely measure the NT indicator and often miss the opportunity to collect and use more information on persons not treated, including their infection status.	Include NT indicator in pre-TAS, coverage evaluation survey and SCT. Standardize recording and analysis of answers to increase understanding on who is not being treated, why and their potential impact on transmission.
Ensuring representation of the NT in data collection	Concern that current tools to measure coverage and infection prevalence are missing persons not treated.	Explore ways to adjust data collection tools to be more inclusive.

## Box 1. Secondary data analysis

- Analyse data from MDA registers that include age, sex and reasons for non-treatment, recognizing this does not capture non-treatment due to lack of access.
  - Explore differences between once-only treatment and never treated, the reasons and how these are affected by age and sex.
- Triangulate coverage evaluation survey (CES) data with MDA treatment registers to determine if recall bias is an issue in CES responses.
- Expand modelling to allow users to include never-treat data.
  - What is the relationship between reported and modelled treatment coverage and the proportion of people never treated? Can we estimate a never-treated proportion from treatment coverage?
  - For modelling based on these data, consider what assumptions would be required for how many people have had 1, 2 or 3 treatments or would be covered in subsequent rounds and evaluate if this is important or whether the dynamics are driven by the never-treated proportion.
- Publish aggregate coverage evaluation survey data analyses that re-analyse existing data based on standard categorizations and exclude those who would have been too young to participate in any MDA.

## Box 2. Operational research priorities

- Measure who was never treated.
  - Analyse whether never-treated people are more likely to be infected, especially in low-prevalence areas, including links to baseline infection intensity.
  - Develop profiles of persons never treated and reasons for not being treated.
  - Develop a stand-alone set of questions to collect data on never treatment, reasons why and proposed solutions.
- Measure reasons why people were never treated.
  - Measure the percentage of never treatment due to intentional (refusal) versus unintentional (access) issues.
  - Determine whether the reasons why people were never treated are similar to the reasons why people were treated once.
- Determine the impact of never treatment.
  - Determine whether people who were never treated are a reservoir of infection.
  - Determine the transmission potential of never-treated populations through modelling.
  - Determine the impact of missing different population groups on infection prevalence, e.g. are those who perceive themselves to be ineligible more likely to be infected than those who refuse treatment for other reasons?
  - Through modelling, determine the appropriate level of never treatment in various groups that should trigger action.
- Define responses to never being treated.
  - Develop and test cost-effective intervention strategies targeted to reasons for never being treated.
  - Explore how much coverage might increase from various programmatic responses to the different elements of why people are never treated.
  - Determine if never treatment is a proxy for primary healthcare access.
    - Explore whether identifying and engaging people who have never been treated can have catalysing effects on health.

## References

[1] World Health Organization. Ending the neglect to attain the Sustainable Development Goals: a roadmap for neglected tropical disease 2021–2030 Geneva: World Health Organization; 2020.

[2] Gabrielli A-F, Montresor A, Chitsulo L, Preventive chemotherapy in human helminthiasis: theoretical and operational aspects. Trans R Soc Trop Med Hyg 2011;105(12):683–93. 22040463 10.1016/j.trstmh.2011.08.013 PMC5576527

[3] Dyson L, Stolk WA, Farrell SH, Measuring and modelling the effects of systematic non-adherence to mass drug administration. Epidemics 2017;18:56–66. 28279457 10.1016/j.epidem.2017.02.002 PMC5340860

[4] Oswald WE, Kepha S, Halliday KE, Patterns of individual non-treatment during multiple rounds of mass drug administration for control of soil-transmitted helminths in the TUMIKIA trial, Kenya: a secondary longitudinal analysis. Lancet Glob Health 2020;8(11):e1418–26. 33069302 10.1016/S2214-109X(20)30344-2 PMC7564382

[5] Stolk WA, Prada JM, Smith ME, Are alternative strategies required to accelerate the global elimination of lymphatic filariasis? Insights from mathematical models. Clin Infect Dis 2018;66(Suppl 4): S260–6. 29860286 10.1093/cid/ciy003 PMC5982795

[6] United Nations. Transforming our world: the 2030 Agenda for Sustainable Development New York: United Nations; 2015.

[7] World Health Organization, World Bank. Tracking universal health coverage: first global monitoring report Geneva: World Health Organization; 2015.

[8] World Health Organization. Preventive chemotherapy: tools for improving the quality of reported data and information. A field manual for implementation Geneva: World Health Organization; 2019.

[9] World Health Organization. Global programme to eliminate lymphatic filariasis: monitoring and epidemiological assessment of mass drug administration Geneva: World Health Organization; 2011.

[10] World Health Organization. Monitoring drug coverage for preventive chemotherapy Geneva: World Health Organization; 2010.

[11] El-Setouhy M, Abd Elaziz K, Helmy H, The effect of compliance on the impact of mass drug administration for elimination of lymphatic filariasis in Egypt. Am J Trop Med Hyg 2007;77(6):1069–73. 18165524 PMC2196399

[12] Krentel A, Damayanti R, Titaley CR, Improving coverage and compliance for mass drug administration for the elimination of LF in two ‘endgame’ districts in Indonesia using micronarrative surveys. PLoS Negl Trop Dis 2016;10(11):e0005027. 27812107 10.1371/journal.pntd.0005027 PMC5094770

[13] Won KY, Beau de Rochars M, Kyelem D, Assessing the impact of a missed mass drug administration in Haiti. PLoS Negl Trop Dis 2009;3:e443. 19707279 10.1371/journal.pntd.0000443 PMC2724684

[14] World Health Organization. Global programme to eliminate lymphatic filariasis: progress report, 2020. Wkly Epidemiol Rec 2021;96(41):497–508.

[15] Niles RA, Thickstun CR, Cox H, Assessing factors influencing communities’ acceptability of mass drug administration for the elimination of lymphatic filariasis in Guyana. PLoS Negl Trop Dis 2021;15(9):e0009596. 34543269 10.1371/journal.pntd.0009596 PMC8452018

[16] Dickson BFR, Graves PM, Aye NN, The prevalence of lymphatic filariasis infection and disease following six rounds of mass drug administration in Mandalay Region, Myanmar. PLoS Negl Trop Dis 2018;12(11):e0006944. 30419025 10.1371/journal.pntd.0006944 PMC6258426

[17] Farrell SH, Truscott JE, Anderson RM. The importance of patient compliance in repeated rounds of mass drug administration (MDA) for the elimination of intestinal helminth transmission. Parasit Vectors 2017;10(1):291. 28606164 10.1186/s13071-017-2206-5 PMC5469187

[18] Krentel A, Damayanti R, Titaley CR, Improving coverage and compliance in mass drug administration for the elimination of LF in two ‘endgame’ districts in Indonesia using micronarrative surveys. PLoS Negl Trop Dis 2016;10(11):e0005027. 27812107 10.1371/journal.pntd.0005027 PMC5094770

[19] Krentel A, Fischer PU, Weil GJ. A review of factors that influence individual compliance with mass drug administration for elimination of lymphatic filariasis. PLoS Negl Trop Dis. 2013;7(11): e2447. 24278486 10.1371/journal.pntd.0002447 PMC3836848

[20] Boyd A, Won KY, McClintock SK, A community-based study of factors associated with continuing transmission of lymphatic filariasis in Leogane, Haiti. PLoS Negl Trop Dis 2010;4(3): e640. 20351776 10.1371/journal.pntd.0000640 PMC2843627

[21] Mathieu E, Direny AN, De Rochars MB, Participation in three consecutive mass drug administrations in Leogane, Haiti. Trop Med Int Health 2006;11(6):862–8. 16772008 10.1111/j.1365-3156.2006.01626.x

[22] Vanamail P, Ramaiah KD, Subramanian S, Pattern of community compliance with spaced, single-dose, mass administrations of diethylcarbamazine or ivermectin, for the elimination of lymphatic filariasis from rural areas of southern India. Ann Trop Med Parasitol 2005;99(3):237–42. 15829133 10.1179/136485905X29666

[23] World Health Organization. Global programme to eliminate lymphatic filariasis: progress report, 2019. Wkly Epidemiol Rec 2020;99(43):509–24.

[24] Sackett D, Haynes R. Compliance with therapeutic regimens Baltimore: Johns Hopkins University Press; 1976.

[25] Anghel LA, Farcas AM, Oprean RN. An overview of the common methods used to measure treatment adherence. Med Pharm Rep 2019;92(2):117–22. 31086837 10.15386/mpr-1201 PMC6510353

[26] Aronson JK. Compliance, concordance, adherence. Br J Clin Pharmacol 2007;63(4):383–4. 17378797 10.1111/j.1365-2125.2007.02893.x PMC2203247

[27] McKay C, Verhagen E. ‘Compliance’ versus ‘adherence’ in sport injury prevention: why definition matters. Br J Sports Med 2016;50(7): 382–3. 26682865 10.1136/bjsports-2015-095192

[28] World Health Organization. Forms for data collection at the peripheral level – Register Available from: https://apps.who.int/neglected_diseases/ntddata/forms/jap/en/Form_register.pdf [accessed 25 September 2023].

[29] Coutts SP, King JD, Pa’au M, Prevalence and risk factors associated with lymphatic filariasis in American Samoa after mass drug administration. Trop Med Health 2017;45:22. 28794687 10.1186/s41182-017-0063-8 PMC5543440

[30] Graves PM, Sheridan S, Fuimaono S, Demographic, socioeconomic and disease knowledge factors, but not population mobility, associated with lymphatic filariasis infection in adult workers in American Samoa in 2014. Parasit Vectors 2020;13:125. 32164780 10.1186/s13071-020-3996-4 PMC7068921

[31] Lau CL, Sheel M, Gass K, Potential strategies for strengthening surveillance of lymphatic filariasis in American Samoa after mass drug administration: reducing ‘number needed to test’ by targeting older age groups, hotspots, and household members of infected persons. PLoS Negl Trop Dis 2020;14(12):e0008916. 33370264 10.1371/journal.pntd.0008916 PMC7872281

[32] Willis GA, Mayfield HJ, Kearns T, A community survey of coverage and adverse events following country-wide triple-drug mass drug administration for lymphatic filariasis elimination, Samoa 2018. PLoS Negl Trop Dis 2020;14(11):e0008854. 33253148 10.1371/journal.pntd.0008854 PMC7728255

[33] Krentel A, Basker N, Beau de Rochars M, A multicenter, community-based, mixed methods assessment of the acceptability of a triple drug regimen for elimination of lymphatic filariasis. PLoS Med 2021;15(3):e0009002. 10.1371/journal.pntd.0009002 PMC7928496 33657090

[34] Silumbwe A, Zulu JM, Halwindi H, A systematic review of factors that shape implementation of mass drug administration for lymphatic filariasis in sub-Saharan Africa. BMC Public Health 2017;17(1):484. 28532397 10.1186/s12889-017-4414-5 PMC5441010

[35] Shuford KV, Turner HC, Anderson RM. Compliance with anthelmintic treatment in the neglected tropical diseases control programmes: a systematic review. Parasit Vectors 2016;9:29. 26813098 10.1186/s13071-016-1311-1 PMC4729159

[36] Setouhy EL, Abd Elaziz KM, Helmy H, The effect of compliance on the impact of mass drug administration for elimination of lymphatic filariasis in Egypt. Am Soc Trop Med Hyg 2007;77(6):1069–73. PMC2196399 18165524

[37] Dickson BFR, Graves PM, Aye NN, Risk factors for lymphatic filariasis and mass drug administration non-participation in Mandalay Region, Myanmar. Parasit Vectors 2021;14(1):72. 33482891 10.1186/s13071-021-04583-y PMC7821648

[38] Campillo J, Awaca-Uvon N, Missamou F, Results from two cohort studies in central Africa show that clearance of *Wuchereria bancrofti* infection after repeated rounds of mass drug administration with albendazole alone is closely linked to individual adherence. Clin Infect Dis 2021;73(1):e176–83. 32856050 10.1093/cid/ciaa1232 PMC8246789

[39] King CL, Weil GJ, Kazura JW. Single-dose triple-drug therapy for *Wuchereria bancrofti*—5-year follow-up. N Engl J Med 2020;382(20):1956–7. 32402169 10.1056/NEJMc1914262 PMC7175637

[40] Jose R, Bougma R, Drabo F, Proxy responses for mass drug administration coverage surveys: the trends and biases when others are allowed to respond. Am J Trop Med Hyg 2021;106(1):268–74. 34695783 10.4269/ajtmh.21-0817 PMC8733507

